# Combination of Nanovectorized siRNA Directed against Survivin with Doxorubicin for Efficient Anti-Cancer Activity in HER2+ Breast Cancer Cells

**DOI:** 10.3390/pharmaceutics14112537

**Published:** 2022-11-21

**Authors:** Sahar Eljack, Emilie Allard-Vannier, Yoann Misericordia, Katel Hervé-Aubert, Nicolas Aubrey, Igor Chourpa, Areeg Faggad, Stephanie David

**Affiliations:** 1 EA6295 Nanomédicaments et Nanosondes, Université de Tours, 37200 Tours, France; 2 Department of Pharmaceutics, Faculty of Pharmacy, University of Gezira, Wad Medani 21111, Sudan; 3 ISP UMR 1282, INRAE, Equipe BioMAP, Université de Tours, 37200 Tours, France; 4 Department of Molecular Biology, National Cancer Institute University of Gezira (NCI-UG), Wad Medani 21111, Sudan

**Keywords:** siRNA, targeted nanovector, HER2+, Doxorubicin, Survivin

## Abstract

According to Globocan 2020, breast cancer is considered one of the most common cancers affecting women and is one of the leading causes of death in over 100 countries. The available classical treatment options do not always give satisfactory outcomes, and some patients develop resistance to these treatments. This study aims to investigate the combination of nanovectorized siRNA directed against anti-apoptotic protein Survivin (siSurvivin) by targeted stealth magnetic siRNA nanovectors (TS-MSN), designed in our lab, with Doxorubicin (DOX), as an option for HER2+ breast cancer treatment. The hypothesis is that the pretreatment of the HER2+ breast cancer cell line SK-BR-3 with siSurvivin will induce apoptosis in the cancer cells and enhance the therapeutic efficacy of DOX, allowing a dose reduction of DOX and hence a reduction of potential side effects. TS-MSN are based on superparamagnetic iron oxide nanoparticles (SPIONs) covalently coupled with a fluorophore sulfocyanine-5 and polyethylene glycol 5000 (PEG_5000_) and functionalized with single-chain variable fragments (scFv) of an antibody targeting the HER2 membrane receptor. These covalently functionalized SPIONs are then complexed via electrostatic interactions with therapeutic siRNA and the cationic polymers, chitosan, and poly-L-arginine. TS-MSN_siSurvivin_ had an average size of 144 ± 30 nm, a PDI of 0.3, and a slightly positive zeta potential value of 10.56 ± 05.70 mV. The agarose gel electrophoresis assay confirmed that the siRNA is well-complexed into TS-MSN without leakage, as no free siRNA was detected. Moreover, siRNA in TS-MSN was protected from RNAse A degradation for up to 6 h at 37 °C. Formulations of TS-MSN with siSurvivin demonstrated in vitro gene knockdown up to 89% in the HER2+ breast cancer cell line SK-BR-3. Furthermore, qRT-PCR confirmed a significant Survivin mRNA relative expression inhibition (about 50%) compared to control siRNA or untreated cells. A combination protocol was evaluated between TS-MSN and Doxorubicin (DOX) for the first time. Therefore, SK-BR-3 cells were pretreated with TS-MSN formulated with siSurvivin at 50 nM for 24 h alone, before a DOX treatment at a concentration of 0.5 µM (corresponding to the IC_50_) was added for 48 h. The MTT cytotoxicity tests, performed after 72 h of treatment, revealed that the combination had a significant synergistic cytotoxic effect on SK-BR-3 cells compared to monotherapies or untreated cells. We confirmed that pretreatment of cells with siSurvivin potentializes the cytotoxic effect of DOX as an alternative approach for treating HER2+ breast cancer. In conclusion, a combination of anti-Survivin siRNA and DOX would be a good alternative in HER2+ breast cancer therapy.

## 1. Introduction

According to Globocan, in 2020, there were 2.3 million women diagnosed with breast cancer and 685,000 deaths globally. As of the end of 2020, there were 7.8 million women alive diagnosed with breast cancer in the past 5 years, making it the world’s most prevalent cancer. Breast cancer mortality changed little from the 1930s to the 1970s. Improvements in survival began in the late 1990s in countries where early detection programs, combined with different treatment modes and explorations of its molecular subtypes, emerged to control the invasive disease [1,2]. About 15–20% of breast carcinomas overexpress human epidermal growth receptor 2 (HER2), which correlates with more aggressive tumor behavior and poor clinical outcome [3].

Treatment options may include surgery, radiation therapy, HER2-directed therapy, and cytotoxic drugs. The best combination of treatments, and the order in which to give them, can vary depending on the situation. Early and locally advanced HER2-positive breast cancer is commonly treated with neoadjuvant chemotherapy such as anthracyclines, taxanes, and cyclophosphamide, in combination with the anti-HER2 monoclonal antibody trastuzumab (Herceptin^®^), with remarkable results [4]. Doxorubicin (DOX), an anthracycline, remains an indispensable option in the clinical treatment of HER2+ breast cancer both in neoadjuvant and adjuvant settings. It mediates its cytotoxic effect through the (i) inhibition of the synthesis of DNA through intercalation, and by inhibition of the topoisomerase II, leading to changes in the chromatin structure, and (ii) generation of free radicals and oxidative damage to biomolecules [5,6,7,8]. However, the nonspecific DOX tissue distribution, inducing resistance in the body, and high hemo/cardiotoxicities limit its applications. In this study, DOX was used as a model chemotherapeutic. Additionally, some women present some resistance to trastuzumab over time. The HER2 overexpression is also correlated with resistance to classical treatments, indicating the need for new specific therapies. One possibility is to use nucleic acid-based therapeutics such as short-interfering RNAs (siRNAs). The therapeutic potential of nucleic acid-based drugs resides in their capability to downregulate overexpressed proteins implicated in tumorigenesis and/or in different cell survival pathways. Recently, gene regulatory-based approaches were presented as a fast-growing area with an increasing number of ongoing clinical trials [9].

siRNAs are 20–25 nucleotides in length metabolized from a large RNA molecule by an endogenous nuclease. The siRNA molecules, in turn, bind to a protein complex, termed the RNA-induced silencing complex (RISC), which unwinds the two strands of RNA molecules, allowing the antisense strand to attach to the targeted RNA molecule, causing effective suppression of gene expression. Both in vitro and in vivo work have demonstrated the therapeutic potential of RNA interference [10]. Naked nucleic acids are unfavorable for systemic delivery because of their inherent limitations, such as negative charge, large molecular weight, size, instability, and difficulties crossing the cell membrane. Furthermore, they are easily degraded by serum endonucleases and are quickly eliminated by renal excretion. In this regard, engineered nanocarriers can stably encapsulate/complex, protect, and selectively deliver therapeutic nucleic acids to tumor tissues. The ideal system should preferably be non-toxic, biodegradable, non-immunogenic, and able to deliver siRNA efficiently to target tumors. More importantly, after tumor tissue-specific delivery, the nano-system should facilitate the intracellular uptake of the pay-loaded nucleic acids and promote their endosomal release/escape into the cytoplasm to induce their gene-regulatory effect [11].

Our team has developed the targeted stealth magnetic siRNA nanovectors (TS-MSN) [12]. The originality of these TS-MSN consists of their theranostic potential, where the diagnostic function is linked to the superparamagnetic iron oxide nanoparticle (SPION) core labeled with a covalently attached near-infrared fluorescent dye for MRI and optical detection *in vivo*, respectively [13]. Additionally, magnetic properties of SPION can be explored with an external magnetic field to target cancer cells, enhance the drug delivery, and induce cell death by hyperthermia [14,15,16]. However, in this study, the theranostic potential of TS-MSN will not be fully explored as the focus of the current works lies on the therapeutic potential of TS-MSN. The synthesis and formulation protocols were previously developed and published. The cores are coated with an organic layer containing several polymers. First, polyethylene glycol (PEG_5000_) is covalently attached to the SPIONs’ surface, providing the nanoparticles’ colloidal stability and immune stealthiness features [17]. PEG is also conjugated with an anti-HER2 single-chain variable fragment (scFv) in an orientation- and sequence-controlled manner to obtain a selective and enhanced targeting of HER2+ breast cancer cells [18]. Finally, these targeted nanoparticles were loaded with siRNA and cationic polymers (chitosan and poly-L-arginine (PLR)) using a previously reported protocol [12,19]. The cationic polymers enable siRNA complexation and the subsequent siRNA endosomal escape to reach the cytoplasmic compartment [12].

The siRNA used for this study is an anti-Survivin siRNA (siSurvivin). Survivin is a member of the inhibitor of apoptosis (IAP) proteins, representing a group of negative regulators of both caspases and cell death. Survivin has multiple functions in cytoprotection, cell death inhibition, and cell cycle regulation, all of which favor cancer cell survival [20,21]. Therefore, Survivin may be a potential target for siRNA-based anti-cancer therapy due to its higher expression levels in breast cancer and other types of cancers [7,15]. Furthermore, Survivin overexpression may be a predictive factor in determining the response to chemotherapy and radiotherapy in breast cancer patients [22,23,24]. Additionally, it has been shown that inhibition of Survivin can restore the sensitivity of cancer cells to anti-cancer agents [25,26].

As mentioned, emerging new treatment modalities are crucial in treating the resistant form of breast cancer. Breast cancer’s dynamic and versatile nature has been a critical challenge for developing efficient and safe therapies. Cancer treatments using a single therapeutic agent, including monoclonal antibodies, chemotherapy, or tyrosine kinase inhibitors, often result in limited clinical outcomes due to tumor heterogeneity and drug resistance. Combination therapies using multiple therapeutic modalities can synergistically elevate anti-cancer activity while lowering doses of each agent, reducing the side effects. Combining two therapeutics with a distinctive mechanism of action in one or two separate doses remains a cornerstone modality in cancer therapy. 

This study aimed to combine nanovectorized siSurvivin via TS-MSN and Doxorubicin (DOX) for efficient HER2+ breast cancer treatment. The hypothesis is that the pretreatment of the HER2+ breast cancer cell line SK-BR-3 with anti-Survivin siRNA will induce apoptosis in the cancer cells and enhance the therapeutic efficacy of DOX, allowing a dose reduction of DOX and hence a reduction of potential side effects. To achieve the goal, TS-MSN were formulated and characterized. The characterization includes the evaluation of TS-MSN physicochemical parameters, their potential to protect siRNA against degradation by RNAse, and their potential to downregulate the Survivin protein and mRNA expression on the HER2+ breast cancer cell line, SK-BR-3. In parallel, the half-maximal concentration (IC_50_) of DOX was determined at different time points from 4 to 72 h on SK-BR-3 HER2+ breast cancer cells. Finally, the in vitro cytotoxicity of a combination treatment between TS-MSN formulated with siSurvivin and DOX was evaluated on the same HER2+ cell line.

## 2. Materials and Methods

### 2.1. Materials 

NHS-PEG-Maleimide (NHS-PEG-Mal, Mw 5000 Da) and sulfocyanine 5 NHS ester were obtained from Rapp Polymer GmbH (Tübingen, Germany) and Lumiprobe (Hannover, Germany), respectively. Poly-L-arginine (MW 15,000–70,000) and high-purity chitosan (MW 110,000–150,000), used for TS-MSN formulation, were provided by Sigma–Aldrich Chemie GmbH (Schnelldorf, Germany). Model siRNA (sequence in Table 1) for physicochemical characterization was purchased from Eurogentec (Angers, France). siRNA against Survivin (sequence in Table 1) was purchased from Sigma–Aldrich Chemie GmbH (St. Quentin Fallavier, France). Doxorubicin hydrochloride was purchased from TEVA Pharmaceutics Ltd. (Puteaux, France). Commercial transfection reagents Lipofectamine^™^ RNAiMAX, Lipofectamine^™^ 3000, and Oligofectamine^™^ were purchased from Life Technologies (Paisley, UK). For gel retardation assays, loading buffer, agarose, and ethidium bromide were provided by Fisher Bioreagents^®^ (Illkirch, France). The 3-(4,5-dimethylthiazol-2-yl)-2,5-diphenyltetrazolium bromide, the MTT reagent, was purchased from Sigma–Aldrich (Saint-Quentin Fallavier, France). Life Technologies (Paisley, UK) supplied all the culture media and supplements for cell culture. RIPA buffer was obtained from Sigma–Aldrich Chemie GmbH (Schnellodorf, Germany). The bicinchoninic acid (BCA) protein assay kit was purchased from Bio-Rad (Hercules, CA). The primary antibody against Survivin (anti-rabbit), glyceraldehyde 3-phosphate dehydrogenase (GAPDH, anti-rabbit), and peroxidase-conjugated secondary antibody (HRP goat to rabbit) were provided by Life Technologies (Paisley, UK). The enhanced chemiluminescence (ECL) kit was purchased from Thermo Pierce (Illkrich-Graffenstaden, France). The Nucleospin^®^ RNA extraction kit was purchased from Macherey-Nagel (Hœrdt, Germany). The RevertAid First-Strand cDNA Synthesis Kit was purchased from Thermo Scientific (Paisley, UK). Takyon MasterMix 2X and primers (sequences in Table 1) were provided by Eurogentec (Seraing, Belgium). Water was obtained from a Milli-Q system (Millipore, Paris, France). All solutions were prepared with deionized water, and all reagents were of analytical grade.

### 2.2. Nanocarrier Synthesis and Formulation

Stealth fluorescent nanoparticles were synthesized using previously developed protocols. Briefly, SPIONs obtained using the Massart protocol were first silanized [16,27]. Then, silanized SPIONs were labeled with the fluorochrome sulfocyanine 5 NHS and functionalized with NHS-PEG-Mal (MW 5000), forming stealth florescent SPIONs [18,28]. In parallel, scFv anti-HER2 was produced in *E. coli* and purified using affinity chromatography [13]. Finally, stealth florescent SPIONs were functionalized with purified scFv, leading to the development of targeted stealth fluorescent SPIONs (TSF-SPIONs) [13,18]. Targeted stealth magnetic siRNA nanovectors (TS-MSN) were prepared based on a protocol previously described by Bruniaux et al. [12,19]. Briefly, siRNA was pre-complexed with poly-L-arginine (PLR), then added to a suspension containing functionalized SPIONs and chitosan using a micropipette, and mixing and vortexing were applied after each addition to obtain a homogenous suspension. The amount of the final targeted functionalized SPIONs (quantified by its iron content) was defined as the iron/siRNA mass ratio and fixed at 10. Chitosan and PLR contents were expressed by the charge ratio (CR) of positive polymer charges to negative siRNA charges. The CR of chitosan/siRNA (CR/CS) and PLR/siRNA (CR/PLR) was set to 30 and 10, respectively. The final siRNA concentration was 50 nM for transfection experiments and 2000 nM for physicochemical characterization.

### 2.3. Nanocarrier Characterization

The mean hydrodynamic diameter (D_H_), polydispersity index (PDI), and zeta potential (ζ) of TS-MSN in suspension were determined using a Malvern Nanosizer ZS (Malvern Instruments, Malvern, UK). Before measurement, the TS-MSN formulation was diluted in NaNO_3_ 0.01 M at a ratio of 1:25 (*v*/*v*) to fix the ionic strength. All the measurements were achieved at 25 °C in triplicate and presented as mean values ± SD.

### 2.4. SiRNA Complexation Assay by Agarose Gel Electrophoresis 

The agarose gel electrophoresis assay was performed to check the complexation of siRNA into the TS-MSN. Samples were prepared to deposit 1.2 µM of siRNA per well. Samples were used in the presence and absence of heparin (final concentration of 3 mg/mL) (Sigma–Aldrich Chemie GmbH, Steinheim, Germany). A loading buffer (2X RNA loading dye, Life Technologies, Paisley, United Kingdom) was added to the samples before their placement in the wells. An agarose gel (1% m/v) was prepared by dissolving agarose (Low-EEO/Multi-Purpose, Acros Organics BV, Geel, Belgium) in Tris-acetate-EDTA (TAE) solution 1X (Acros Organics BV, Geel, Belgium), containing 0.01% (*v*/*v*) ethidium bromide (EtBr), to visualize free siRNA. After the deposition of the samples on the agarose gel, the migration was conducted in TAE 1X buffer for 15 min at 150 V. The gels were visualized by UV-imaging using the EvolutionCapt software on a Fusion-Solo.65.WL imager (Vilbert Lourmat, Marne-la-Vallée, France).

### 2.5. SiRNA Protection against Enzymatic Degradation 

To analyze the siRNA protection against RNAse degradation, TS-MSN (at an initial siRNA concentration of 2.5 µM) were incubated with an aqueous ribonuclease A solution (1.2 µg/mL Sigma–Aldrich Chemie GmbH, Schelldorf, Germany) at a ratio of 2:1 for 30 min, 2, 4, and 6 h at 37 °C. Afterward, ribonuclease A was inactivated by heating the suspensions at 70 °C for 30 min. An equivalent amount of free siRNA was incubated for 30 min and used as a positive control to check the ribonuclease A activity to analyze the amount of free siRNA. Samples were diluted in water (Milli-Q system, Millipore, Paris, France) or aqueous heparin sodium solution (10 mg/mL) Sigma–Aldrich Chemie GmbH, Schnelldorf, Germany) and mixed with the loading buffer (agarose gel loading dye 6X) to deposit 20 mol ofsiRNA per well on 1% agarose gel containing ethidium bromide. With its strong negative charge, heparin is used as a control to displace complexed siRNA from TS-MSN. A 150 V voltage was applied for about 15 min in a Tris/acetate/EDTA buffer (TAE 1X, 40 mM acetate, EDTA 1 mM, pH 7.6). Gels were visualized and analyzed with EvolutionCapt software on a FusionSolo.65.WL imager (Vilbert Lourmat, Marne-la-Vallée, France).

### 2.6. Cell Culture

The SK-BR-3 human breast carcinoma cell line with HER2 overexpression was purchased from Cell Lines Service (CLS Eppelheim, Germany). SK-BR3 cells were grown at 37 °C/5% CO_2_ in Dulbecco’s Modified Eagle Medium (DMEM) supplemented with 10% fetal calf serum and 1% penicillin/streptomycin. The culture medium was changed every 48 h, and the cells were harvested using trypsin as soon as 80% confluency was reached.

### 2.7. SiRNA Transfection 

Here, 3 × 10^5^ or 5 × 10^5^ of SK-BR-3 cells per well were seeded in a 6-well plate for protein extraction for further use in the Western blot experiment. For RNA extraction for qRT-PCR, 12-well plates were seeded with 3 × 10^5^ cells 24 h before transfection. On the day of transfection, anti-Survivin siRNA and control (Ctrl.) siRNA were formulated in TS-MSN and diluted in OptiMEM^™^ serum-free medium for 4 h to obtain a 50 nM siRNA final concentration inside each well. Afterward, the SK-BR-3 culture medium was added (1:1 *v*/*v*). Oligofectamine^™^, Lipofectamine^™^ 3000, and Lipofectamine^™^ RNAiMAX (Invitrogen, Thermo Fisher Scientific (Paisley, UK)) were used as control transfection agents and were formulated according to their manufacturer’s recommendations with anti-Survivin siRNA to obtain the same siRNA concentrations. Cells were treated with the prepared suspensions and maintained in normal growth conditions for an additional 68 h. The level of Survivin protein and mRNA expressions were determined by Western blotting and qRT-PCR, respectively, as described below. 

### 2.8. Protein Extraction and Western Blot

After transfection of cells with siSurvivin via TS-MSN and commercial transfection agents for 72 h, the transfected cells were washed with cold PBS, and total proteins were extracted on ice using RIPA buffer supplemented with protease inhibitor. After 15 min, a centrifugation step was performed (10,000 G, 4 °C) to collect the supernatant; then, protein concentrations were determined using the bicinchoninic acid (BCA) protein assay kit. The cell lysate (15–30 µL protein for each sample) was boiled for 5 min in sodium dodecyl-sulfate (SDS) buffer and subjected to polyacrylamide gel electrophoresis (PAGE). The proteins were transferred to the nitrocellulose membrane using an iBlot^®^ 2 dry blotting system (Thermo Fisher Scientific, Illkirch, France). After blocking with 5% nonfat milk at room temperature for 1 h, the membrane was incubated with the primary antibody against Survivin (anti-rabbit, 1:1000) or glyceraldehyde-3-phosphate dehydrogenase (GAPDH, anti-rabbit, 1:1000/1:2000) at 4 °C overnight. After incubation with the peroxidase-conjugated secondary antibody (HRP goat to rabbit, 1:1000), the protein was visualized via an enhanced chemiluminescence (ECL) kit on a Fusion-Solo.65.WL imager (Vilbert Lourmat, Marne-laVallée, France) using EvolutionCapt software.

### 2.9. Design of Experiments

To optimize the conditions of Western blot experiments, the design of experiments was used in the form of a complete 2² factorial design. The independent process parameters chosen were: (A) the number of cells seeded on 6-well plates and (B) the serum concentration of the complete cell culture medium that was added after 4 h of incubation with the nanovectors. Each parameter was studied at the two levels listed in Table 2. The levels were chosen to provide a maximal design space and still enable feasible Western blot experiments. One response value, the Survivin protein expression, was analyzed by Western blot. For an easier interpretation, the results were transformed into numerical estimated values.

### 2.10. RNA Extraction and QRT-PCR Analysis

According to the manufacturer’s recommendation, total RNA was extracted using the Nucleospin^®^ RNA extraction kit. The quantity of isolated RNA was measured by Nanodrop^™^ (Thermo fisher scientific, Illkirch, France), and the samples were diluted to a final concentration of 200 ng/µL. According to the manufacturer’s recommendation, reverse transcription (RT) for cDNA synthesis was performed using the RevertAid First-Strand cDNA Synthesis Kit. Then, a qPCR mix was prepared for each gene (target gene: Survivin, reference gene: GAPDH) using the Takyon MasterMix 2X, reverse and forward primers for the selected gene, and RNAse-free water. Then, 2 µL of cDNA (diluted 1/20 with nuclease-free water before use) was mixed with 13 µL of qPCR Mix in a 96-well plate. All samples were deposited in triplicate with each couple of primers. The plate was then heated at 95 °C for 3 min to activate the Taq polymerase, then 40 cycles at 95 °C for 10 s (denaturation) and at 60 °C for 40 s (annealing/extension) were performed using the Bio-Rad thermocycler C300 Touch. CFX Manager software (Bio-Rad, Hercules, CA, USA) was used to analyze the samples. Non-treated cells were used as a negative control, and cells treated with the commercial transfection reagent Lipofectamine^TM^ RNAiMAX (Invitrogen, Carlsbad, CA, USA) formulated with siSurvivin, abbreviated as Lipofectamine, were used as a positive control. Survivin’s normalized, relative gene expression was calculated using the ΔΔCq method [29]. Briefly, the number of quantification cycles (Cq) was determined for the target gene (Survivin) and the reference gene (glyceraldehyde 3-phosphate dehydrogenase (GAPDH)) for each sample using the CFX Manager software. ΔCq was then determined by normalization of the Survivin gene expression to GAPDH gene expression (=Cq (Survivin) – Cq (GAPDH)). ΔΔCq was determined by averaging the ΔCq and then normalizing it to that of the non-treated cells (ΔΔCq = ΔCq (sample) – ΔCq (non-treated cells)). Finally, the ΔΔCq was then exponentially transformed to find the ΔΔCq expression, which was equal to 2^- ΔΔCq^ [29].

### 2.11. Cytotoxicity of TS-MSN Components

Cell viability was evaluated by the MTT assay. SK-BR-3 cells were seeded at 5 × 10^3^ cells/well in a 96-well plate for 24 h. Cells were treated with a mixture of (a) Ctrl. siRNA + SPIONs, (b) Ctrl. siRNA + PLR + chitosan, and (c) TS-MSN formulated with Ctrl. siRNA. Non-treated cells and cells treated with H_2_O_2_ at 20 mM were used as the negative and positive controls, respectively. The culture medium was then replaced by the mixture of 190 µL of fresh medium and 10 μL of an aqueous solution of MTT (yellow) (5 g/L) and incubated for 4 h at 37 °C. The medium/MTT mixture was removed, and 200 μL of DMSO was added to each well. The plate was agitated until the all-formed formazan crystal (purple) was homogenous and completely dissolved in the solvent. The absorbance was measured at 540 nm using KC-junior V1.40 software on a BIOTEK EL 800-microplate reader. The viability percent was calculated as the percentage of absorbance of the study group over the control group (positive control = cells treated with hydrogen peroxide, H_2_O_2_, and negative control = untreated cells incubated with culture medium). 

### 2.12. Cytotoxicity of DOX (IC_50_ Determination) 

DOX initial half-maximum concentration (IC_50_) studies at different time points: 4, 24, 48, and 72 h, were carried out to determine the optimal DOX concentration to be tested in combination with therapeutic siRNA. The DOX range was from 0.025 up to 10 µM, following the same steps as the MTT cytotoxicity assay described above. 

### 2.13. Cytotoxicity of The Combination of TS-MSN Formulated with SiSurvivin and DOX 

The treatment regimen of separate delivery of DOX and TS-MSN with siSurvivin was evaluated on the SK-BR-3 cell line using the MTT cytotoxicity assay. The treatment plan began with cell treatment with siSurvivin formulated in TS-MSN at a concentration of 50 nM. Then, 24 h post-siRNA transfection, cells were treated with Doxorubicin at 0.5 µM until 72 h. Forty-eight hours after the DOX treatment and seventy-two hours after siRNA treatment, cell viability was evaluated by the MTT assay, as described above. All samples were kept at 37 °C and 5% CO_2_ during treatment. 

### 2.14. Statistical Analysis 

Data are expressed as the mean ± SD of the variables. All the *p*-values and IC_50_ values were calculated using GraphPad PRISM7 software.

## 3. Results and Discussion

### 3.1. Formulation and Physicochemical Characterization of TS-MSN

**Figure 1:**
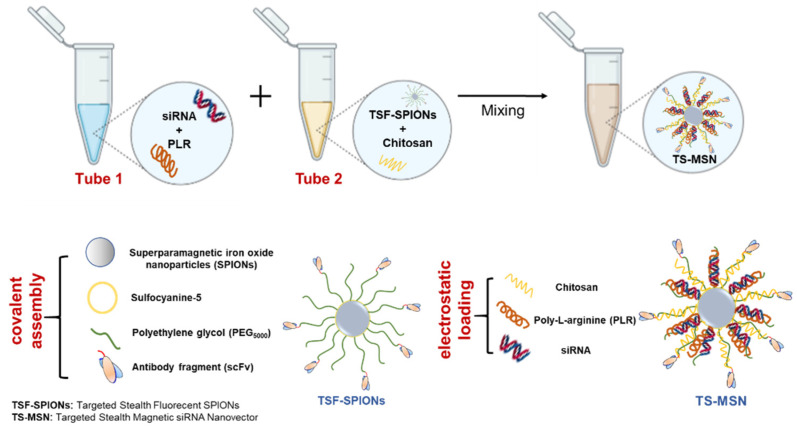
Schematic illustration of the TS-MSN formulation protocol.

TS-MSN were formulated according to a previously developed protocol [8]. Briefly, siRNA was precomplexed with poly-L-arginine (tube 1) and targeted stealth fluorescent SPIONs (TSF-SPIONs) were mixed with chitosan (tube 2) in equal volumes before mixing the content of both tubes. All components were mixed in a well-defined ratio to produce self-assembled nanovectors through electrostatic interaction and form TS-MSN (Figure 1). The formulation process mainly depends on tuning the formulation parameters to assure the reproducibility of the physicochemical characteristics. Careful optimization of the charge ratio, the formulation components’ molar ratio, and the technique’s specificity, including micropipette mixing and vortexing, is an essential precaution during the formulation process of TS-MSN [19,30].

**Figure 2:**
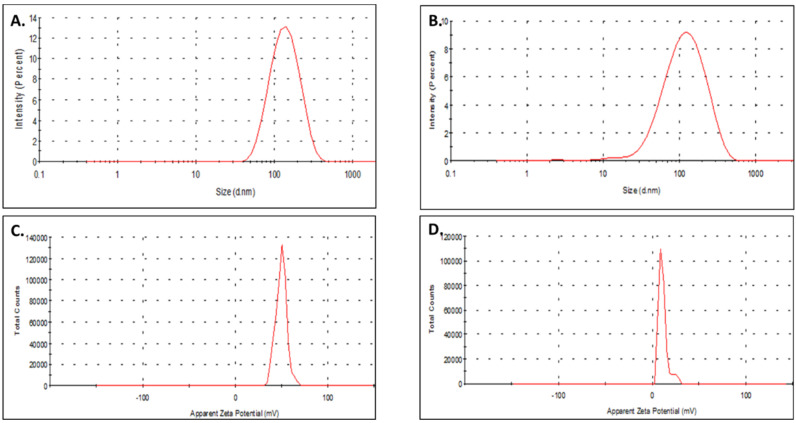
Representative size (**A**,**B**) and zeta potential (**C**,**D**) distribution for TS-MSN_siCtrl_ (**A**,**C**) and TS-MSN_siSurvivin_ (**B**,**D**). Mean values and SD are presented in Table 3.

The hydrodynamic diameter of TS-MSN formulated with Ctrl. siRNA was 131 ± 18 nm, while the polydispersity index (PDI) was 0.306 ± 0.016, and zeta potential was 12.44 + 7.34 mV (Figure 2, Table 3). Furthermore, the values were quite similar for formulations with different siRNA sequences (Ctrl. siRNA and siSurvivin, Figure 2 and Table 3), indicating that the siRNA sequence did not influence the physicochemical parameters of TS-MSN. These values were in accordance with previously obtained results, where the TS-MSN vectorized with siRNA had a hydrodynamic size of 157 ± 22, PDI of 0.3 ± 0.04, and slightly positive zeta potential of 17 ± 4 mV [12]. The size of the TS-MSN in this report was less than 200 nm, with a slightly positive surface charge in an aqueous buffer, which is considered favorable for nanoparticle intravenous administration. The functionalization of the layer by single-chain variable fragment (scFv) anti-HER2 was shown to favor a selective uptake of the nanovectors by HER2-overexpressed cell lines [12]. Therefore, we conclude that our formulation is simple and reproducible, with adequate physico-chemical parameters for an intravenous administration.

**Figure 3:**
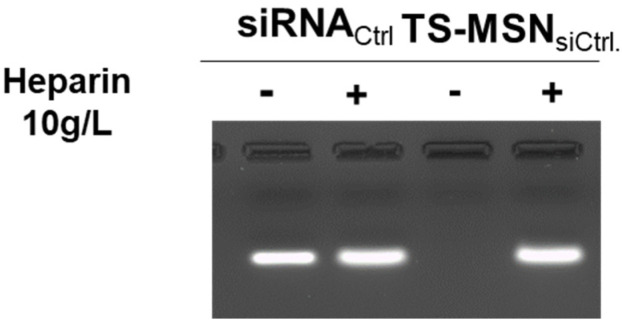
Gel retardation assay demonstrating the siRNA complexation in TS-MSN. siRNA formulated in TS-MSN in the presence (+) or absence (-) of heparin compared to naked siRNA. Lanes without heparin show free siRNA amount, and lanes with heparin show total siRNA amount in the sample. TS-MSN: targeted stealth magnetic siRNA nanovectors.

To check the loading of siRNA into the nanoparticles, a gel electrophoresis experiment based on heparin displacement was performed. An aqueous free/naked siRNA solution was used as a control, and samples of TS-MSN formulated with siRNA were incubated in the absence and presence of heparin (Figure 3). Heparin was added to displace the siRNA from the nanocarriers and hence to visualize the free de-complexed siRNA. As seen in Figure 3, free siRNA can interact with ethidium bromide and yield a fluorescent band. TS-MSN showed no free siRNA as no fluorescence was visible, indicating that siRNA was well-protected by TS-MSN from the intercalation of ethidium bromide. In previous work, the N/P ratio between siRNA and polymers was optimized in order to complex the entire amount of nucleic acids. 

**Figure 4:**
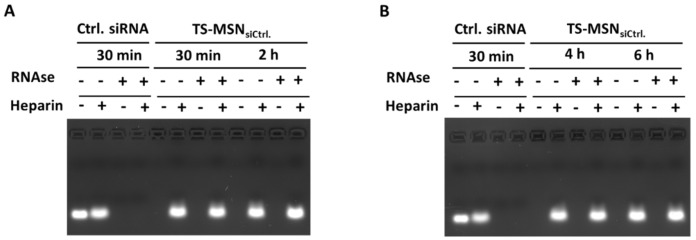
Gel retardation assay demonstrating the siRNA protection against RNAse degradation. siRNA formulated in TS-MSN (**A**) for 30 min and 2 h and (**B**) for 4 and 6 h, in the presence (+) or absence (-) of RNAse A and/or heparin compared to naked non-vectorized siRNA incubated for 30 min. Lanes without heparin show free siRNA amount, and lanes with heparin show total siRNA amount in the sample. TS-MSN: targeted stealth magnetic siRNA nanovectors.

To check the further protection of siRNA in TS-MSN against enzymatic degradation, TS-MSN were incubated with RNAse A. Agarose gel electrophoresis was performed at different time points (Figure 4). Free, non-vectorized siRNA in solution served as a control. Samples of TS-MSN were incubated in the presence and absence of RNAse A and/or heparin for 30 min, 2, 4, and 6 h. As previously mentioned, heparin was added to displace siRNA from the nanocarriers and to visualize complexed siRNA. Naked siRNA was completely degraded within 30 min by RNAse A as no fluorescence band was seen (Figure 4A,B). Conversely, in the absence of RNAse A and heparin, no fluorescence intensity was seen for TS-MSN, indicating complete protection of siRNA in the developed nanovectors. Up to 6 h of incubation, in the presence and absence of RNAse A, lanes with heparin had the same fluorescence intensity of free siRNA for TS-MSN, indicating no siRNA degradation up to 6 h. The protection of the siRNA in the TS-MSN will allow siRNA to withstand the in vitro cellular conditions. Previous reports in our lab confirmed that TS-MSN internalized into different types of breast cancer cells within 4 h after incubation [12]. Furthermore, all those results demonstrated that the developed nano-system could fully complex and protect the loaded nucleic acid inside the nanovectors. This should predict the nanoparticles’ overall stability and contribute to their suitability for potential use via the parenteral route.

### 3.2. The Downregulation Efficiency of TS-MSN on HER2+ Breast Cancer Cells 

**Figure 5:**
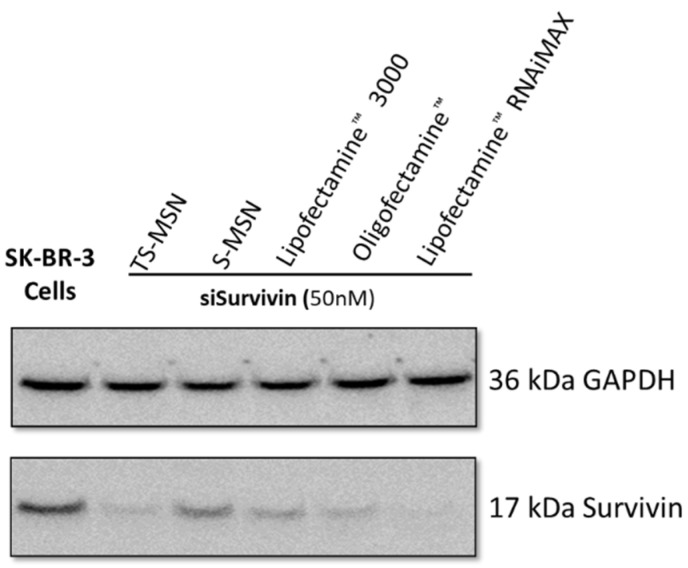
Western blot image of Survivin protein expression on SK-BR-3 cells transfected with TS-MSN, S-MSN, and commercial transfection agents formulated with siSurvivin (50 nM) compared to non-treated cells. TS-MSN: targeted stealth magnetic siRNA nanovectors, S-MSN: stealth magnetic siRNA nanovectors, GAPDH: glyceraldehyde 3-phosphate dehydrogenase.

To investigate the efficacy of TS-MSN as a gene carrier, SK-BR-3, a HER2-overexpressed breast cancer cell line, was used. SK-BR-3 cells were incubated with TS-MSN and S-MSN (nanovectors without scFv for active HER2 targeting) formulated with siSurvivin (50 nM) for 72 h and compared to different commercially available transfection reagents (Figure 5). The used commercial transfection reagents contain lipid subunits that can form liposomes in an aqueous environment, which entrap the transfection payload, such as siRNA, to form lipoplexes. The results demonstrated that various formulations could produce a Survivin protein downregulatory effect to various degrees. TS-MSN had 89% Survivin protein inhibition, and S-MSN around 25%, confirming the HER2 targeting by TS-MSN compared to non-targeted S-MSN in SK-BR-3 cells. This finding was partially consistent with the previous report by Bruniaux et al., where the treatment with TS-MSN produced a significant Survivin protein downregulation efficacy of up to 90% compared to about 70% after treatment with S-MSN. Nevertheless, the results were obtained using another HER2+ breast cancer cell line, BT-474 [12]. The commercial transfection agents Lipofectamine^™^ 3000, Oligofectamine^™^, and Lipofectamine^™^ RNAiMAX downregulate the Survivin protein expression by up to 50%, 65%, and 97%, respectively. That is coherent, as Lipofectamine^™^ RNAiMAX was recently developed especially for siRNA transfection, while Lipofectamine^™^ 3000 was designed to transfect DNA-based therapeutics rather than siRNA-based treatment. Oligofectamine^™^ is considered a first-generation commercial transfection agent. Interestingly, our TS-MSN produced a remarkable effect compared to the tested formulations. For further experiments, the commercial transfection agent Lipofectamine^™^ RNAiMAX was used.

**Figure 6:**
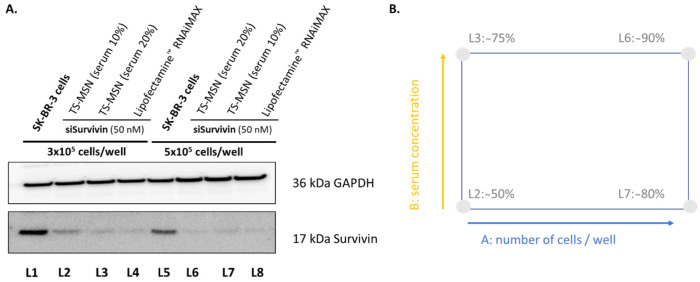
Optimization of the transfection conditions for Western blot experiments using a 2² factorial design. (**A**) Western blot result of Survivin protein inhibition. (**B**) Schematic representation of the results to analyze the 2² factorial design. Values of Survivin protein inhibition are estimated visually from (**A**). TS-MSN: targeted stealth magnetic siRNA nanovectors, GAPDH: glyceraldehyde 3-phosphate dehydrogenase.

To ensure that the transfection conditions for Western blot experiments were optimal, a 2² factorial design was used. According to the previous studies obtained from different results in our lab, the period between the treatment and the analysis was fixed at 72 h to have enough time to inhibit the protein of interest (analysis time), and the duration of incubation with the nanovectors was 4 h in serum-free cell culture medium (treatment time). Non-treated SK-BR-3 cells and cells treated with the commercial transfection agent Lipofectamine^™^ RNAiMAX formulated with siSurvivin were used as a negative and positive control, respectively. After 4 h, the culture media was replaced by complete culture media supplemented with serum for the normal growth of the SK-BR-3 cells. The siSurvivin concentration was fixed at 50 nM. The analyzed factors were: (i) the number of cells deposited per well and (ii) the serum concentration in the culture medium between 4 and 72 h after cell transfection with our nanovectors. Western blot results (Figure 6A) showed that all TS-MSN formulations could induce Survivin protein downregulation but to different degrees. To facilitate the interpretation of the DOE experiment, the inhibition of Survivin expression was quantified visually from the WB images (Figure 6B).

The results indicated that the transfection efficiencies varied between 50% and 90%. Both factors A and B positively impacted the protein downregulation, i.e., the protein downregulation increased when factors A or B increased. Factor A (number of cells/well) seemed to have a more significant impact than factor B (serum concentration) on protein downregulation. There was also an interaction between both factors as the protein downregulation increased depending on whether the second factor was set at the high or the low value. Both factors should be set at a high level to obtain maximum transfection efficiency. With these conditions, the protein downregulation with TS-MSN was similar to that of the positive control Lipofectamine^™^ RNAiMAX. As seen in Figure 6, increasing the number of cells and serum concentration led to the best Survivin protein downregulation. That is attributed to the fact that an adequate number of cells is needed for better transfection efficiency. Generally, 70–90% confluency for adherent cells or 5 × 10^5^ to 2 × 10^6^ cells per/mL for suspension cells at the time of transfection provides good results. Too few cells will cause the cell culture to grow poorly without cell-to-cell contact. The actively divided cells take up the foreign nucleic acid drugs better than quiescent cells, which should be considered when optimum transfection efficiency is needed [31,32]. On the other hand, for optimal transfection conditions, cells should be incubated with a serum-free medium during the first 4 h of treatment to avoid serum interference with our nanovectors. As cells usually cannot withstand a serum-free condition for a long time, a complete culture medium supplemented with an extra serum concentration of 20% could promote healthy cellular division to nourish the cells after such stressful conditions. 

**Figure 7:**
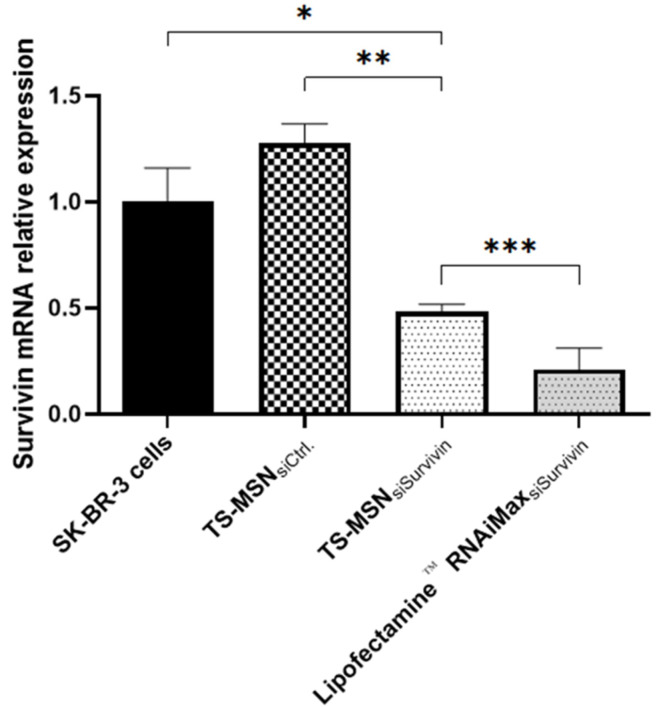
qRT-PCR results of Survivin-mRNA relative expression on SK-BR-3 cells treated with TS-MSN and Lipofectamine^™^ RNAiMAX vectorized with control and anti-Survivin siRNA (50 nM). Data are presented as mean ± SD. * *p* < 0.05, ** *p* < 0.01, *** *p* < 0.001 (*p*-value ≤ 0.05 is considered statistically significant). TS-MSN: Targeted stealth magnetic siRNA nanovectors.

To further investigate TS-MSN efficacy, the relative expression of Survivin mRNA was determined by qRT-PCR in SK-BR-3 cells after treatment with TS-MSN formulated with siSurvivin. Results are shown in Figure 7. Untreated cells and TS-MSN with Ctrl. siRNA served as negative controls, while the commercial transfection agent Lipofectamine RNAiMAX™ formulated with siSurvivin served as a positive control for transfection. The results indicated that the cells treated with the positive control showed significant downregulation of the Survivin mRNA relative expression (0.210 ± 0.010). The expression of Survivin mRNA in the TS-MSN group with siSurvivin (0.482 ± 0.036) was higher than that in the positive control group (*p*-value = 0.0114). Still, it was significantly decreased (*p*-value = 0.0002) compared to the negative control group (TS-MSN with Ctrl. siRNA, 1.276 ± 0.092) or untreated cells (*p*-value = 0.005), indicating a successful downregulation of the Survivin mRNA expression. Arami et al. developed a nanoparticle with a magnetic core (Fe_3_O_4_) covered by polyethylene glycol and lactide. This structure was then covered by chitosan and branched polyethyleneimine (PEI). siSurvivin was then electrostatically loaded on these nanoparticles. They studied their nanovectors on the HER2(-) breast cancer cell line MCF-7. The real-time PCR results showed a Survivin mRNA expression inhibition of 0.4, which was somewhat similar to what we obtained with our system [33]. These results suggest that TS-MSN formulated with siSurvivin induces efficient downregulation of Survivin protein and mRNA expression in the HER2+ breast cancer cell line SK-BR-3. 

### 3.3. In Vitro Cytotoxicity of TS-MSN, Doxorubicin, and a Combination of Both on HER2+ Breast Cancer Cells

**Figure 8:**
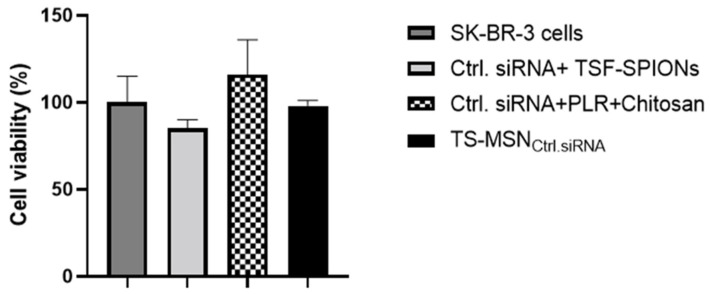
Cell viability of the SK-BR-3 cell line treated either with a mixture of control (Ctrl.) siRNA and TSF-SPIONs, a mixture of Ctrl. siRNA, poly-l-arginine (PLR), and chitosan, or TS-MSN_ctrl. siRNA_. Data are presented as the mean ± SD. TS-MSN: Targeted stealth magnetic siRNA nanovectors, TSF-SPIONs: targeted stealth fluorescent superparamagnetic iron oxide nanoparticles.

This study aimed to use TS-MSN in combination with Doxorubicin (DOX). The cytotoxicity of gene carriers is one of the main disadvantages of nanoparticle-based therapeutics’ delivery and needs to be considered. Many developed nanocarriers have been proven to have high transfection efficiency with high cytotoxicity. This is the case for the commercial transfection agent Lipofectamine^™^ RNAiMAX. Therefore, the first step was to demonstrate the safety of TS-MSN formulated with Ctrl. siRNA on SK-BR-3 cells using the MTT assay. To investigate the cytotoxicity of the components, SK-BR-3 cells were also treated with a mixture of: (a) ctrl. siRNA and TSF-SPIONs, (b) ctrl. siRNA, PLR, and chitosan, and (c) TS-MSN formulated with ctrl. siRNA. Untreated SK-BR-3 cells were used as a control. As shown in Figure 8, all groups had no significant inhibition effect on cell proliferation as the cell viability in each group was above 85%. These results indicate that the cytotoxic effect of TS-MSN mainly depends on the complexed siRNA rather than the TS-MSN carrier or its components and that these nanovectors can be further used for in vivo experiments.

**Figure 9:**
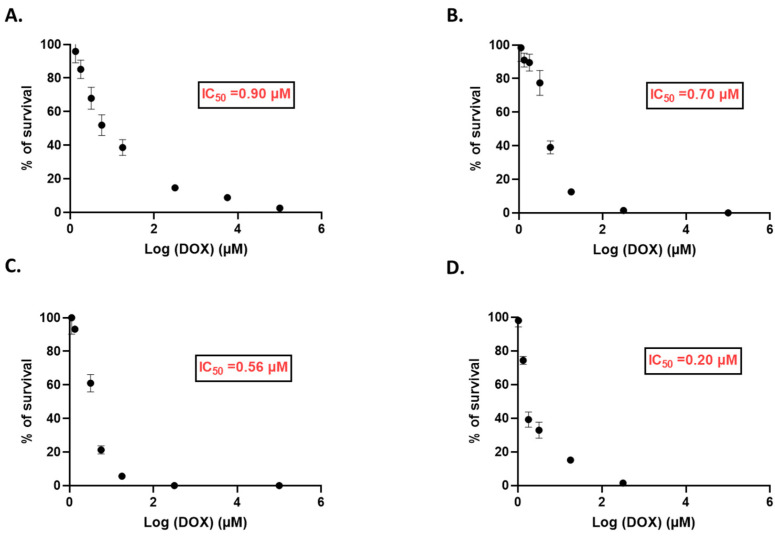
IC_50_ values of Doxorubicin on SK-BR-3 cells, determined at different time points: (**A**) 4 h, (**B**) 24 h, (**C**) 48 h, and (**D**) 72 h using the MTT cytotoxicity assay.

The second step was to determine the DOX IC_50_ at various time points from 4 up to 72 h. Therefore, different concentrations of DOX were tested from 0.025 to 10 µM on SK-BR-3 cells. The DOX IC_50_ values are shown in Figure 9. DOX treatment of 4, 24, 48, and 72 h had an IC_50_ of 0.90, 0.70, 0.56, and 0.20 µM, respectively, showing a time concentration-dependent cytotoxicity. Schindler et al. reported an IC_50_ equal to 0.683 ± 0.287 µM after DOX treatment for 48 h on SK-BR-3 cells [34]. Moreover, Nguyen et al. revealed that DOX treatment in the same cell line for 48 h had an IC_50_ of 0.64 µM, although their cells were treated differently [35]. Nevertheless, we could achieve approximate values to what is reported in the literature. These IC_50_ values allow us to adapt the DOX concentration used for the combination protocols with TS-MSN and serve as reference guides.

**Figure 10:**
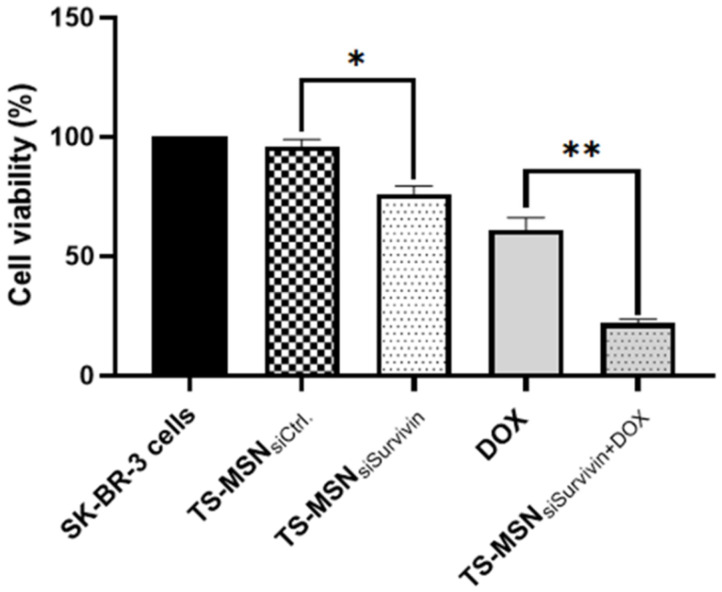
SK-BR-3 cells’ viability after treatment with the combination of TS-MSN and DOX. TS-MSN were formulated either with Ctrl. siRNA or anti-Survivin siRNA and were incubated at a concentration of 50 nM for 72 h. DOX has been added 24 h post-TS-MSN treatment at an IC_50_ concentration of 48 h (0.5 µM). Data are presented as the mean ± SD. * *p* = 0.0015, ** *p* = 0.0001 (both *p*-values were statistically significant as they are <0.05). TS-MSN: Targeted stealth magnetic siRNA nanovectors, DOX: Doxorubicin.

The last step was to combine the individual delivery of TS-MSN formulated with siSurvivin (TS-MSN_siSurvivin_) and DOX in SK-BR-3 cells. The cells were treated with TS-MSN with either Ctrl. or siSurvivin at a concentration of 50 nM for 72 h. We hypothesized that a pre-regulation of Survivin protein with siRNA might potentiate the DOX anti-cancer effect in HER2+ breast cancer cells. Then, 24 h post-siRNA treatment, the cells were treated with DOX at IC_50_ (0.5 µM) for 48 h. The MTT cell viability assay was carried out after 72 h of treatment. Figure 10 shows that the combination of TS-MSN_siSurvivin_ and DOX induced a significant synergistic effect (*p* < 0.05). The cell viability was 22% ± 2%, compared to 61% ± 5% and 76% ± 3.44% with the monotherapies DOX or TS-MSN_siSurvivin_, respectively. Additionally, cells treated with TS-MSN_siSurvivin_ significantly increased cellular toxicity compared to TS-MSN with Ctrl. siRNA (cell viability 96.00% ± 2.92%, *p* < 0.05). In the literature, many studies of separate delivery of siRNA and anti-cancer drugs conclude that pretreatment with siRNA targeting different proteins involved in cancer cell survival, growth, and death, eventually leads to synergizing the anti-cancer efficacy and reduction of the drug concentration used, which contributed to a better therapeutic outcome with fewer side effects [36,37]. Xu et al. reported that the pretreatment with siSurvivin for 24 h increased DOX’s sensitivity in hypoxic laryngeal cancer AMC-HN-8 and Hep-2 hepatocellular carcinoma cell lines. The IC_50_ of DOX in AMC-HN-8 cell lines had a significant decline from 3.84 ± 0.23 to 2.67 ± 0.14 µg/mL [36]. Li et al. showed that Survivin’s stable knockdown by siRNA inhibits tumor cell growth and angiogenesis in breast and cervical cancers, leading to an increased apoptotic rate in response to different pro-apoptotic stimuli, such as Doxorubicin or TNF-alpha [38]. These findings are promising and could provide an alternative option for the selective treatment of HER2-overexpressed breast cancer.

## 4. Conclusion and Perspectives 

This study evaluated targeted stealth magnetic siRNA nanovectors (TS-MSN) for their physicochemical properties and cellular activity alone or in combination with the widely used anthracycline Doxorubicin as a promising approach for in vitro application in a HER2+ breast cancer model. The main conclusions are: (1) The TS-MSN formulation is simple and reproducible, with appropriate characteristics suitable for a potential parenteral administration. TS-MSN formulated with different siRNA sequences and with different batches had similar physico-chemical characteristics. The size was less than 200 nm, with a slightly positive surface charge, and TS-MSN protected siRNA against RNAse A for up to 6 h. (2) The Survivin downregulation efficacy of TS-MSN_siSurvivin_ was moderate at the mRNA level (about 50%), but high at the protein level (about 90%). The gene silencing effect of TS-MSN_siSurvivin_ was similar to the commercial transfeaction agent Lipofectamine^™^ RNAiMAX. However, this commercial transfection agent is not suitable for in vivo experiments. TS-MSN had the further advantage that its component toxicity was lower than that of Lipofectamine^™^ RNAiMAX. (3) A pretreatment with TS-MSN_siSurvivin_ followed by a non-vectorized DOX treatment resulted in a synergistic cytotoxic effect on HER2+ breast cancer cells. The combination of TS-MSN and DOX showed interesting results, enabling further exploration in an in vivo HER2+ breast cancer model, and could be an interesting alternative for the selective treatment of HER2-overexpressed breast cancer.

## Figures and Tables

**Table 1:** Sequences of siRNAs and primers used for qRT-PCR.

**siRNA**	**Sequence**
Ctrl. sense	5′-GGAAGAUCAUAAUGGACAG[dT][dT]-3′
Ctrl. antisense	5′-CUGUCCAUUAUGAUCUUCC[dT][dT]-3′
Survivin sense	5′-GUCUGGACCUCAUGUUGUU[dT][dT]-3′
Survivin antisense	5′-AACAACAUGAGGUCCAGAC[dT][dT]-3′
**primer**	**Sequence**
GAPDH reverse	5′-AGTTGTCATGGATGACCTTGG-3′
GAPDH forward	5′-CAAAAGGGTCATCATCTCTGC-3′
Survivin reverse	5′-CCGGACGAATGCTTTTTATG-3′
Survivin forward	5′-GCCCAGTGTTTCTTCTGCTT-3′

Ctrl.: control, GAPDH: glyceraldehyde 3-phosphate dehydrogenase, [dT]: deoxyribonucleotides.

**Table 2:** Factors (A, B) and levels of the 2² factorial design.

Experiment	A: Number of Cells	B: Serum Concentration
1	300,000	10%
2	500,000	10%
3	300,000	20%
4	500,000	20%

**Table 3:** Physicochemical characteristics of TS-MSN with control and anti-Survivin siRNAs.

	Hydrodynamic Diameter (nm)	Polydispersity Index	Zeta Potential (mV)
TS-MSN_siCtrl._	131 ± 18	0.302 ± 0.038	+12.44 ± 07.34
TS-MSN_siSurvivin_	144 ± 30	0.288 ± 0.030	+10.56 ± 05.70

TS-MSN: Targeted stealth magnetic siRNA nanovectors, Ctrl.: control.
